# Empowering therapeutic antibodies with IFN-α for cancer immunotherapy

**DOI:** 10.1371/journal.pone.0219829

**Published:** 2019-08-08

**Authors:** Jun Guo, Yu Xiao, Ramesh Iyer, Xin Lu, Marc Lake, Uri Ladror, John Harlan, Tanushree Samanta, Medha Tomlinson, Gail Bukofzer, Cherrie Donawho, Alex Shoemaker, Tzu-Hsuan Huang

**Affiliations:** 1 Oncology Discovery, AbbVie Inc., North Chicago, United States of America; 2 Global Protein Sciences, AbbVie Inc., North Chicago, United States of America; 3 Genomics Research Center, AbbVie Inc., North Chicago, United States of America; 4 Global Biologics, AbbVie Bioresearch Center, Worcester, United States of America; University of Pécs Medical School, HUNGARY

## Abstract

Type 1 IFNs stimulate secretion of IP-10 (CXCL10) which is a critical chemokine to recruit effector T cells to the tumor microenvironment and IP-10 knockout mice exhibit a phenotype with compromised effector T cell generation and trafficking. Type 1 IFNs also induce MHC class 1 upregulation on tumor cells which can enhance anti-tumor CD8 T cell effector response in the tumor microenvironment. Although type 1 IFNs show great promise in potentiating anti-tumor immune response, systemic delivery of type 1 IFNs is associated with toxicity thereby limiting clinical application. In this study, we fused tumor targeting antibodies with IFN-α and showed that the fusion proteins can be produced with high yields and purity. IFN fusions selectively induced IP-10 secretion from antigen positive tumor cells, which was critical in recruiting the effector T cells to the tumor microenvironment. Further, we found that treatment with the anti-PDL1-IFN- α fusion at concentrations as low as 1 pM exhibited potent activity in mediating OT1 CD8^+^ T cell killing against OVA expressing tumor cells, while control IFN fusion did not exhibit any activity at the same concentration. Furthermore, the IFN-α fusion antibody was well tolerated *in vivo* and demonstrated anti-tumor efficacy in an anti-PD-L1 resistant syngeneic mouse tumor model. One of the potential mechanisms for the enhanced CD8 T cell killing by anti-PD-L1 IFN fusion was up-regulation of MHC class I/tumor antigen complex. Our data supports the hypothesis of targeting type 1 IFN to the tumor microenvironment may enhance effector T cell functions for anti-tumor immune response.

## Introduction

Programmed cell death-1(PD-1) is a checkpoint receptor protein expressed by activated T cells that binds to the programmed cell death ligands (PD-Ls) expressed by antigen presenting cells and tumor cells, resulting in the inhibition of T-cell proliferation, survival, and effector functions [1] Blocking antibodies against the PD-1/PD-L1 show clinical benefit (e.g. pembrolizumab has demonstrated overall response rates of 21%-34% in several tumor types [2]). However, for most solid tumor types excluding melanoma, only a small portion of patients typically respond to PD-1/PD-L1-targeted therapies. Therefore, improved treatment options are needed to increase patient response and survival [3].

Previous studies have shown that interferon-alpha (IFNα), a member of the type 1 interferon family, is a pleiotropic cytokine. IFNα has multiple anti-tumor properties including direct tumor cell killing [4] and stimulating host immune cells including dendritic cells [5] and CD8^+^ T cells [6]. IFN-α is approved by FDA for the treatment of multiple hematologic malignancies, and solid cancers (melanoma, renal cell carcinoma and Kaposi’s sarcoma). However, recombinant IFN-α alone, which stimulates cancer-fighting immune effector cells, is not well tolerated when administered systemically [7, 8]. To overcome this challenge, as well as to explore mechanisms for improving current PD-1/PD-L1 immunotherapies, we have developed a novel approach by fusing IFN- α to an anti-PD-L1 antibody to direct IFN-alpha activity specifically to the tumor microenvironment. Our data demonstrated that the anti-PD-L1 IFN fusion protein enhanced tumor antigen specific CD8^+^ T cell killing *in vitro* and was effective in an anti-PD-L1 resistant syngeneic mouse tumor model *in vivo*. The potential mechanisms of the anti-PD-L1 IFN fusion efficacy have also been investigated.

## Materials and methods

### IFN-alpha constructs and fusion proteins

DNA sequences encoding the fusion proteins were synthesized and cloned into our proprietary pHybE (US Patent 8187836 B2) vector. The pHybE expression vector utilizes an EF-1α promoter and an OriP origin of replication derived from Epstein-Barr virus (EBV). The mature murine IFN alpha 1 sequence (NP_034632, 24–189 amino acid) was cloned at the C-terminal of the heavy chain of cetuximab or anti- mouse PD-L1[9] linked by a Ser-(Gly)_4_-Ser sequence. HEK-293-6E cells, a suspension adapted human embryonic kidney-293-based cell line, stably expressing the Epstein–Barr virus nuclear antigen (EBNA1), were transfected with plasmid DNA encoding the heavy chain (HC) and light chain (LC) of the fusion protein. For a 3L expression, a 5L flask containing cells at 1.2 x 10^6^ cells/ml were grown in Freestyle 293 Expression medium (ThermoFisher) at a temperature of 37˚C, with 8% CO_2_, and shaking at 80 rpm. For transfection, 1.5 mg of DNA in 2:3 ratio (HC:LC) was mixed with 6 ml of 1 mg/ml pH 7.0 PEI solution (Polysciences) in a volume of 150 ml Freestyle medium. After 10 minutes of incubation, the mixture was added to the cells. Tryptone N1 (Organotechnie) in Freestyle medium was added to the flask at 24 hours post transfection for 0.5% final concentration. The conditioned medium was harvested 9–11 days post transfection by centrifugation at 16K x G for 10 minutes, followed by clarification through an AcroPak 500 0.8/0.45 μm filter capsule (Pall), and sodium azide was added from a 1M stock to a concentration of 5 mM. The conditioned media was stored at 4˚C until purified. The cell culture supernatant containing fusion protein was loaded onto a MabSelect SuRE (GE Life Sciences) column, and washed with 5 column volumes of PBS. The protein was eluted by step elution with 50 mM glycine, 50 mM NaCl pH 3.5 in 10 column volumes. The protein was collected in 7 ml fractions, and neutralized with 0.5 M sodium phosphate buffer pH 8.8. The neutralized fractions containing fusion protein were characterized with analytical SEC, SDS-PAGE gel, and mass spec before pooling. The protein was formulated in PBS, and stored at -80 ˚C.

### Cell lines and reagents

Human colorectal adenocarcinoma cell line (NCI-H747), HEK-293 and LL/2 murine lung adenocarcinoma cell line were obtained from ATCC. MC38 (mouse colon 38) cell line was obtained from National Cancer Institute, and the resource of GEO human colon cancer cell line has been described previously [10]. All cell lines were verified as mycoplasma free using MycoAlert detection kit (Lonza, #LT07). Murine T cells were isolated from spleens using T cell isolation kit from Miltenyi Biotec. The transwells for cell migration assay was purchased from Corning, NY 14831. Anti-CD3 and anti-CD28 antibody were purchased from BD Bioscience.

### IP-10 assay and expression in human tumor samples

PD-L1 positive tumor cells were treated with different IFN fusions for 72h, and IP-10 expression from MC38, LL/2, GEO and NCI-H747 cells was determined using Meso-Scale technology (V-PLEX ELISA based assay). Expression of IP-10 and CD3D in tumor clinical specimen were downloaded from Oncomine Powertool by pooling multiple published datasets using Affymetrix U133p2 or U133A microarrays, all data sets were log-transformed and median-centered per array, and standard deviations were normalized to one per array.

### Transwell migration assays with activated T cells

Mouse T cells were isolated from C57B spleens by negative selection using magnetic beads (Miltenyi Biotec). The selected cells were cultured with anti-CD3/anti-CD28 monoclonal antibody coated plates for 48 hours. These cells were loaded into the top chamber of transwell inserts (5.0 μm pore size, Costar). In the bottom well, culture supernatant from MC38 cells treated with different antibodies was added. Plates were incubated at 37 ˚C for 4 h; the contents of the lower chamber were collected and the fold increase compared with control of CD3+ T cells in the bottom chamber was determined by CellTiter-Glo assay (Promega).

### Flow cytometry

Cells were blocked using the block agent from BioLegend, washed with PBS, and stained with Live/Dead reagent (Invitrogen) and antibodies specifically recognizing mouse H-2Kb (Biolegend, Cat#116506) and mouse H2Kb bound to OVA peptide (Biolegend, Cat#141604) for 20 minutes on ice and washed twice. FACS acquisition was done on the BD Canto II using FlowJo software (BD Bioscience) for analysis.

### *In Vitro* antigen-specific T cell killing of tumor cells in co-culture

Chicken ovalbumin (NM_205152) cDNA were cloned into pCDH-EF1-MCS-IRES-Puro cDNA cloning and expression vector (System Biosciences, CA). HEK293T cells were seeded at 80% confluency in 6-well plates. One day after seeding, the cells were transfected in Gibco Opti-MEM I Reduced Serum Medium (Life Technologies, USA) using the construct expression vector, FuGENE 6 (Promega), and Misson Lentiviral Packaging Mix (Sigma). The medium was changed after 6 hours of incubation at 37 °C and 5% CO_2_. The viral supernatants were collected at 48 hours after transfection. Harvested viral supernatants were filtered through a 0.22-μm membrane and stored at −80°C. MC38 and LL/2 cells were transduced with the harvested lentiviral particles. Each cell line was then separately transduced in the presence of 4 μg/mL of polybrene (Sigma-Aldrich, USA) with the lentiviral particles. The medium was changed two day after the lentiviral transduction. After six day transduction, the medium changes included 2 μg/mL of puromycin. Mouse tumor cell lines (LL/2-OVA and MC38-OVA) stably expressing chicken ovalbumin have been generated after the puromycin selection. We evaluated antigen-specific T cell killing using IncuCyte Zoom (Essen BioScience Ltd, UK) and Cell Titer Glo for cell viability assays (Promega). Murine OT1 CD8^+^ T cells and control CD8^+^ T cells were isolated from spleen using pan T cell isolation kit with negative magnetic selection (Miltenyi Biotec). T cells were counted using a Vi-CELL Counter. To enable direct analysis of the target tumor cell proliferation, Cells were transfected using nuclear restricted RFP kit (Essen BioScience, UK). Two thousand MC38-OVA or LL/2-OVA NucLight Red tumor cells were seeded in 96 well plates overnight, and required number of OT1 CD8^+^ T cells or control CD8^+^ T cells per well were added in second day, and images were captured every 4 hours in IncuCyte Zoom as well as analysis using integrated software(Essen BioScience, UK). For cell viability assays, co-culture cells were treated in 96 well plates with antibodies for 5 days followed by a Cell Titer Glo assay (Promega).

### *In vivo* efficacy studies

All animal studies were conducted in accordance with the guidelines established by the internal Institutional Animal Care and Use Committees (IACUC) at AbbVie, INC (North Chicago, IL). AbbVie is committed to the internationally accepted standard of the 3Rs (Reduction, Refinement, Replacement) and adhering to the highest standards of animal welfare in the company’s research and development programs. Animal studies were approved by AbbVie's Institutional Animal Care and Use Committee or Ethics Committee. Animal studies were conducted in an AAALAC accredited program where veterinary care and oversight was provided to ensure appropriate animal care. A total of 5 x 10^4^ LL/2 cells were inoculated subcutaneously into the right flank of 14-week-old female CB6F1 mice (10 mice per group) on Day 0. Mice were dosed IP with anti-TeTx, anti-PD-L1 or anti-PD-L1-IFN-α on days 7, 11 and 15 post inoculation. Tumor length (L) and width (W) were measured via electronic caliper and the volume was calculated according to the following equation: V = L x W2/2 using Study Director version 3.1 (Studylog Systems, Inc., South San Francisco). Tumor measurements were regularly performed one to three times per week. After cells were inoculated, animals were monitored once daily by trained staff to watch for tumor growth, a combination of clinical signs, including; ascites (fluid on abdomen), reluctance to ambulate, anorexia, rapid respiration, dehydration, hunched posture, scruffy coat and weight loss. Mean group body weights was taken at least once per week. The percentage max. weight loss (day) was the day following inoculation of tumor cells until the end of study, that the greatest percentage of weight loss, relative to the initial weight of the cage of mice, is recorded.

### qPCR analysis

All mice tumors were collected in fresh conditions, and the tumor cells were isolated using Tumor Dissociation Kit (cat# 130-095-929) following the Miltenyi Biotec manufactures instructions. The filtered tumor cells were centrifuged for 5 minutes at 350xg, and the cells were re-suspended in cell washing buffer (BioLegend, cat# 420201). RNA was isolated by using RNeasy Mini Kit (cat# 74104 from Qiagen). Reverse transcription was carried out via a two-step invitrogen’s protocol (cat# 18080–051). qPCR assays were carried out with Applied Biosystems QuanStudio 7 Flex system using TagMan fast advanced master mix (cat# 4444557) and CXCL10 from ThermoFisher (cat# mm00445235 m1). Data normalization was carried out using the GAPDH as a housekeeping gene.

## Results

### Production and characterization of anti-PD-L1- IFN-α

**Fig 1:**
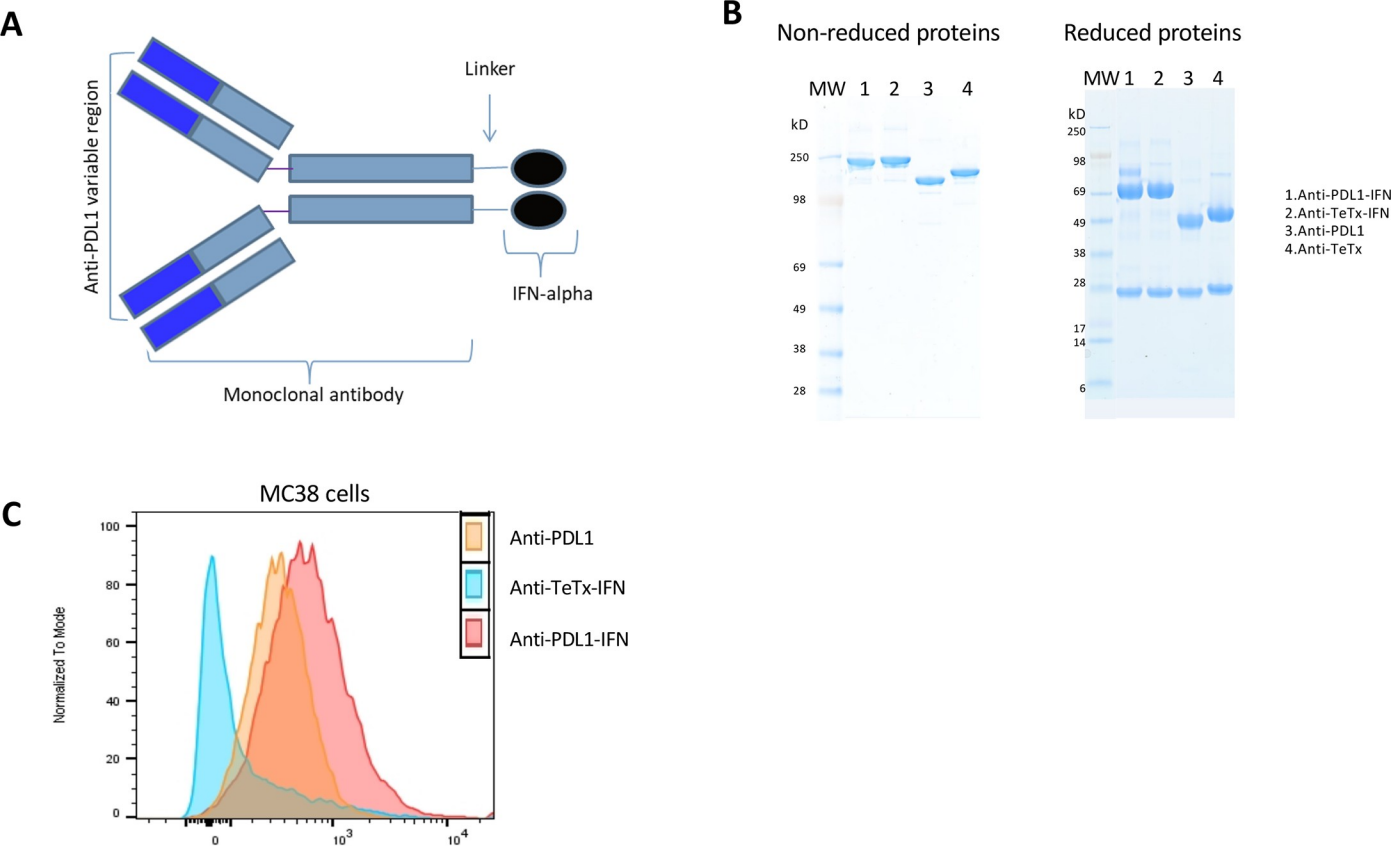
Production and characterization of IFN-alpha fusion proteins. Anti PD-L1-IFN, Anti-TeTx-IFN fusions, and the corresponding control IgGs were produced in HEK-293-6E cells. The cells were transfected with heavy and light chain plasmids using PEI as the transfection reagent. Purity of the protein was analyzed by SDS-PAGE. **(A)** Schematic diagram of of IFN-alpha fusion proteins and control. **(B)** SDS-PAGE analysis of non reduced and reduced purified fusion proteins and control. **(C)** Binding of mAbs and mAb-IFN-alpha to MC38 cells. After binding to the indicated antibodies, cells were probed with APC-conjugated antibody, and analyzed by FACS.

PD-L1 is variably expressed in tumor tissues [11], and type 1 IFNs play critical roles in tumor control [12]. To evaluate the effectiveness of using IFN fusion to target cancer cells, IFN-α fusion proteins have been generated. The N-terminus of murine IFN-alpha was fused to the Ser-(Gly)_4_-Ser linker of the C-terminus of the heavy chain of anti-PD-L1 or anti-TeTx (Fig 1A). The fusion proteins were produced in HEK-293-6E cells. Purity of the protein was analyzed by SDS-PAGE. Under nonreducing conditions, the molecular weight of anti-PD-L1-IFN-α was approximately 210 kDa. After treatment with beta-mercaptoethanol, the fusion protein migrated as heavy chains attached to IFN-α (about 75 kDa) and light chains (about 25 kDa, Fig 1B). The binding of the IFN fusion proteins to murine PD-L1 was evaluated by FACS analysis against the murine PD-L1-expressing MC38 cell line. The anti-PD-L1-IFN-α retains the antigen-binding ability similar to the non-conjugated anti-PD-L1 antibody (Fig 1C).

### Antibody-IFN Fusion selectively stimulates IP-10 induction from antigen-positive tumor cells

**Fig 2:**
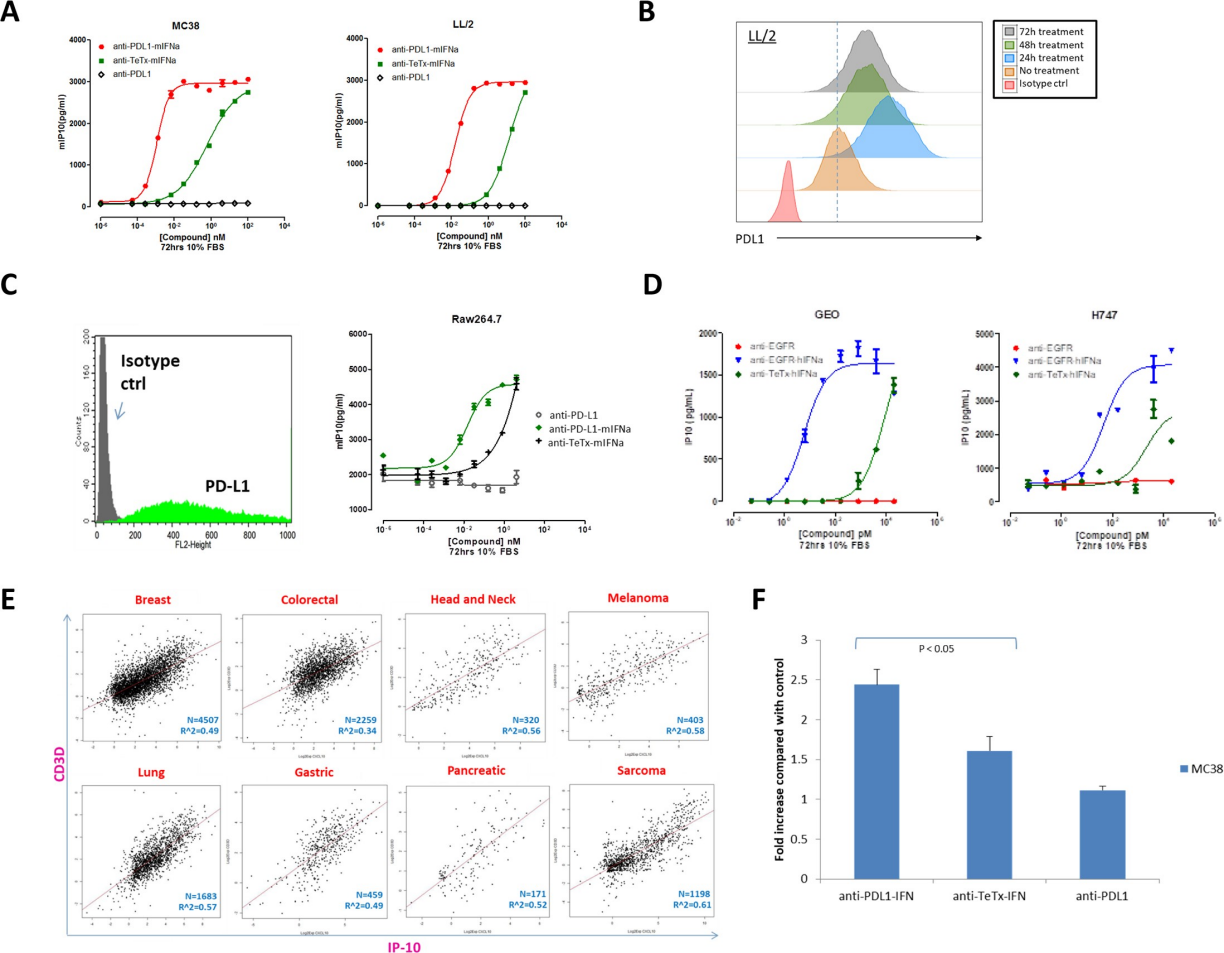
IFN fusions selectively induced IP-10 secretion from antigen positive tumor cells and enhanced T cell infiltration *in vitro*. **(A)** Mouse PD-L1 positive tumor cells (MC38 and LL/2) were treated with different IFN fusions at the indicated concentrations for 72 hours and the IP-10 concentration in the supernatant was measured using IP-10 ELISA assay. **(B)** LL/2 tumor cells were cultured *in vitro* and treated with anti-PDL1-IFN fusion protein (1pM). The expression of PDL1 was measured by flow cytometry at 24h (blue), 48h (green) and 72h (grey) after treatment. PDL1 expression without any fusion protein treatment shown in orange and isotype control shown in red. **(C)** Raw264.7 cells were probed with anti-PD-L1 PE-conjugated antibody and analyzed by FACS (left panel). Raw264.7 cells were treated with different IFN fusions at the indicated concentrations for 72 hours and the IP-10 concentration in the supernatant was measured using IP-10 ELISA assay (right panel). **(D)** Human EGFR positive tumor cells (Geo and H747) were treated with different IFN fusions at the indicated concentrations for 72 hours and the IP-10 concentration in the supernatant was measured using IP-10 ELISA assay. **(E)** IP-10 expression correlates with T cell marker CD3D expression in human tumors. Standardized expression data was downloaded from the public domain by Oncomine, and all of them are p-value < 0.0001. **(F)** Murine T cells were isolated from spleen using negative magnetic selection. 2x10^5^ activated T cells (in RPMI + 0.1%FCS media) were placed into the top chamber of the transwell (5 μm pore size) and 300μl of culture supernatant from MC38 cells treated with different fusion proteins as indicated was placed in the bottom chamber. Cells were allowed to migrate for 4 hours and then cells from the bottom chamber were harvested and counted by CellTiter-Glo assay.

It has been reported that IP-10 is a chemokine specific for effector T cell chemoattraction induced by IFN-α stimulation [13, 14]. To assess if the anti-PD-L1-IFN-α fusion was able to induce IP-10 activity, we compared the activity of antibody alone with IFN-α fusion proteins. MSD assays measuring IP-10 induction were performed on murine PD-L1-expressing cancer cell lines, and although the nontargeted anti-TeTx-IFN-α fusion can induce IP-10 secretion from antigen positive tumor cells, the tumor targeting IFN-α was able to significantly increase IP-10 production at the same concentrations (Fig 2A). IFN-α treatment can increase PD-L1 expression [15] and consistently, we observed that the anti-PD-L1-IFN-α fusion upregulated PD-L1 expression in the mouse cancer cell line, which could potentially enhance IFN-α targeting with the anti-PD-L1 fusion approach (Fig 2B).

In addition to the cancer cell lines, tumor infiltrating immune cells including macrophages express PD-L1 [16]and we showed that PD-L1 targeting IFN-α was able to induce more IP-10 production than the nontargeting anti-TeTx-IFN-α fusion in a PD-L1 positive macrophage cell line (Fig 2C). It is worth mentioning that compared with the recombinant IFN-α, the anti-PD-L1-IFN-α exhibited better IP-10 induction at the lower concentrations potentially through PD-L1 engagement (S1 Fig). This result suggested that the tumor targeting IFN-α may be biologically active at the lower concentrations in the tumors with antigen expression. In addition to anti-PD-L1-IFN-α, we also generated an anti-EGFR-hIFN-α fusion protein and likewise, targeting human IFN-α to EGFR positive human cancer cells (Geo and H747) demonstrated superior IP-10 induction compared with controls (Fig 2D). Further, to examine human IP-10 levels related to T cell infiltration within patient tumors, expression data for IP-10 and CD3D in clinical tumor specimens were examined from multiple published datasets. The analysis suggested that IP-10 gene expression was significantly correlated with the level of T cell infiltration in multiple human tumors (Fig 2E). To examine if IP-10 induction leads to migration of activated T cells, murine activated T cells were placed into the top of transwell chambers and culture supernatant from MC38 cells treated with different fusion proteins was placed in the bottom chamber (Fig 2F). T cells from the bottom chamber were harvested and counted by CellTiter-Glo assay after 4 hours of transwell incubation. CD3+ T cell transmigration increased by 2 fold after anti-PD-L1-mIFN-α treatment compared with anti-TeTx-IFN-α treatment or anti-PD-L1 alone (Fig 2F). Taken together, these data demonstrate selective IP-10 induction from antigen positive cancer cell lines using targeted IFN fusions, and MC38 cancer cells treated with anti-PD-L1-mIFN-α induced better migration of activated T cells compared with the control IFN fusion or antibody alone.

### Targeted IFN fusion enhances OT-1 T cell killing activity against OVA expressing cancer cells

**Fig 3:**
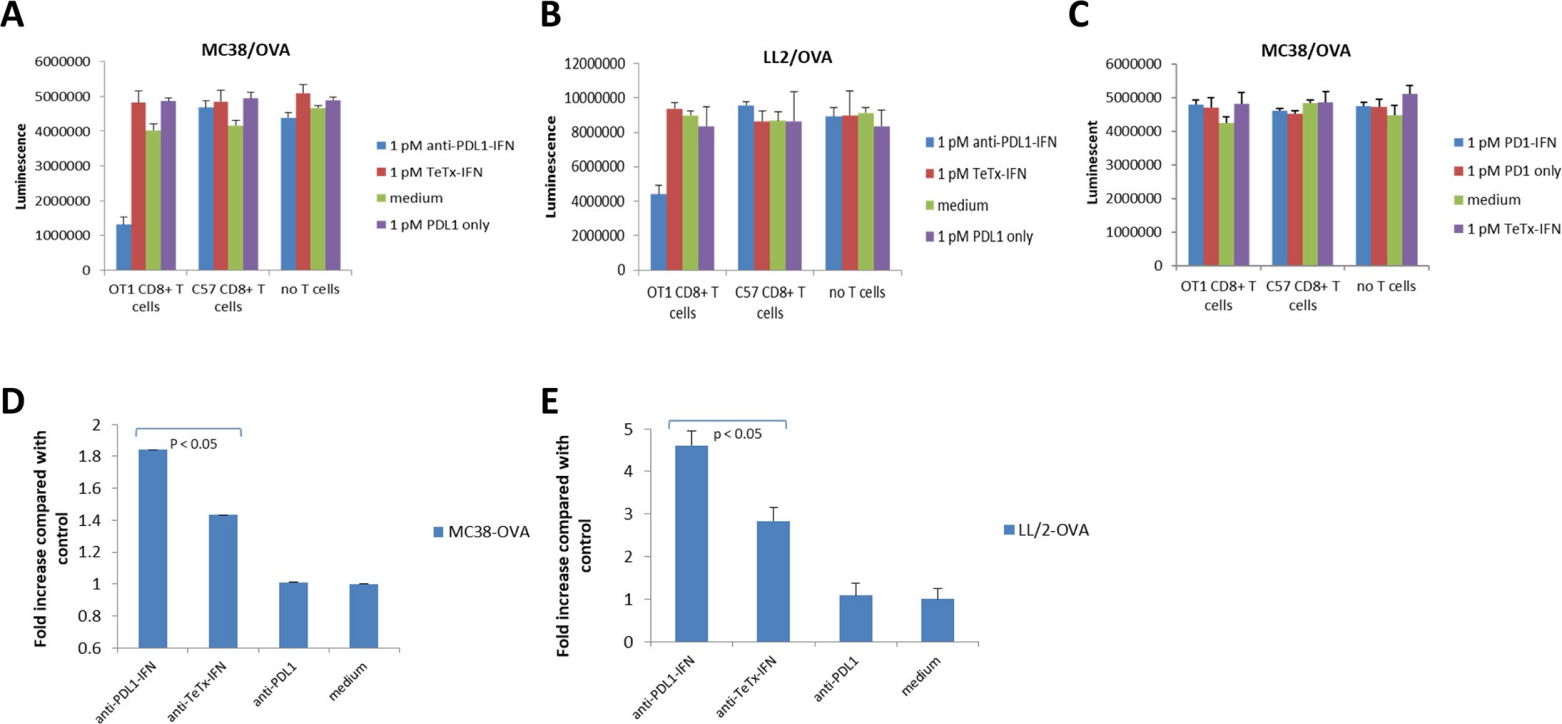
IFN-α fusions enhance antigen-specific T-cell mediated killing of tumor cells. **(A-C)** Two thousand MC38-OVA or LL/2-OVA cells were seeded in 96 well plates overnight. The next day, six thousand murine OT1 CD8^+^ T cells or naïve C57B CD8^+^ T cells were added. Co-cultured cells were treated with the indicated fusion proteins or antibodies for 5 days followed by a Cell Titer Glo assay. **(D-E)** MC38-OVA and LL/2-OVA cells were treated with anti-PDL1 monoclonal antibody, anti-TeTx-IFN fusion and anti-PDL1-IFN fusion at 1 pM. After 48 hours, H2-K^b^/OVA complex expression was measured by FACS analysis.

To investigate whether treatment with targeted IFN fusion proteins can enhance tumor antigen-specific T cell killing activity, we generated chicken ovalbumin over-expressing cell lines (LL/2-OVA and MC38-OVA), and evaluated antigen-specific T cell killing using CellTiter Glo assays. The transgenic OT1 T cell receptor recognizes ovalbumin residues OVA_257-264_ in the context of MHC class I H2-K^b^ and can be used to study the response of CD8^+^ T cells to antigen. CD8^+^ OT-1 T cells were purified from the spleen of OT-I TCR transgenic mice. As a negative control, CD8^+^ T cells purified from the spleen of C57B mice were used. We observed that OT1 and control T cells exhibited insignificant killing effect against OVA positive cancer cells at sub-optimal ratio (T:E = 1:3). Anti-PD-L1-IFN-α but not control fusion protein at 1 pM exhibited strong activity in enhancing the OT1 killing against MC38-OVA or LL2-OVA cells, whereas the fusion protein showed no effect in the killing activity of control CD8 T cells (Fig 3A and 3B). Additionally, anti-PD-L1-IFN-α at 1 pM exhibited no activity in OT1 killing against the parental cancer cell line (S2 Fig), suggesting that the anti-PD-L1-IFN-α mediated effect was antigen-dependent. Both anti-TeTx-IFN-α and recombinant IFN-α showed activity in enhancing the OT1 killing at 1 nM but not at the lower concentrations (10 and 100 pM) in this assay (S3 Fig), suggesting that the PD-L1 targeting IFN-α may be more effective than non-targeting IFN-α in the tumors with PD-L1 expression. Targeting IFN-α to the tumor cells but not the OT-1 T cells was necessary for the enhanced OT-1 killing against OVA expressing cancer cells since an anti-PD-1-IFN-α fusion at 1pM showed no activity in this assay (Fig 3C). Together, these data demonstrates that delivery of IFN-α by anti-PD-L1 enhanced CD8^+^ T cell killing *in vitro*. In order to understand the mechanisms of the enhanced CD8^+^ T cell killing activity after the anti-PD-L1-IFN-α treatment, MC38-OVA and LL/2-OVA cells were treated with anti-PD-L1 antibody, anti-TeTx-IFN fusion and anti-PD-L1-IFN fusion at 1 pM. After 48 hours, H2-K^b^/OVA complex expression was measured by FACS analysis. H2-K^b^/OVA complex was up-regulated after the treatment of IFN-alpha fusions and the anti-PD-L1-IFN-α induced more H2-K^b^/OVA complex expression than other control proteins (Fig 3D and 3E). Although anti-TeTx-IFN-α at 1 pM induced modest increase of H2-K^b^/OVA complex expression, we did not observe a significant OT-1 killing with 1pM anti-TeTx-IFN-α suggesting that a higher level of H2-K^b^/OVA complex expression was necessary to sensitize the OT-1 T cells in our killing assay. These data suggest that up-regulation of H2-K^b^/OVA complex was one potential mechanism for the enhanced CD8^+^ T cell killing observed by treatment with the anti-PD-L1-IFN-α fusion.

### Anti-tumor efficacy in an anti-PD-L1 resistant syngeneic mouse tumor model

**Fig 4:**
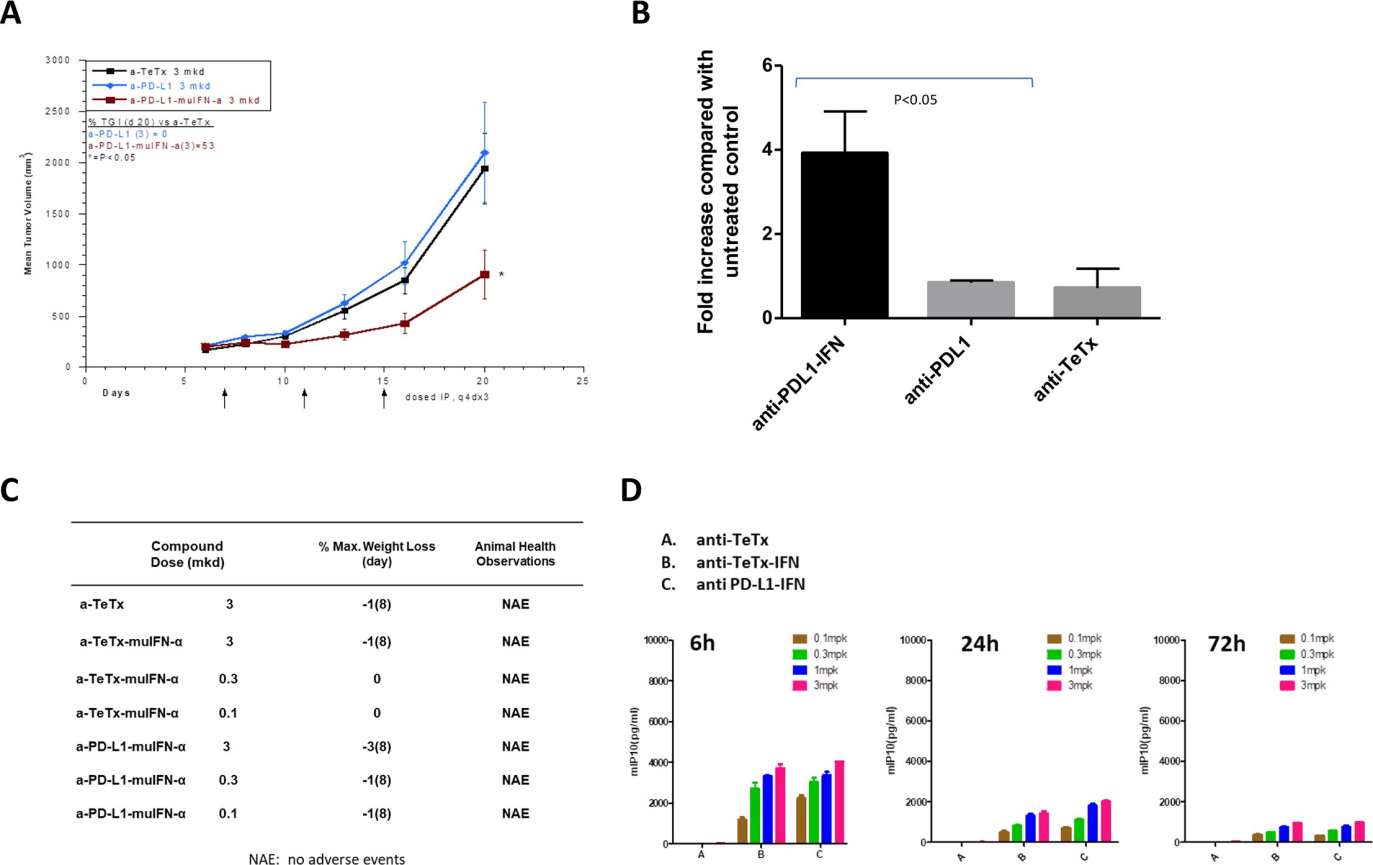
Anti-tumor efficacy in an anti-PD-L1 resistant syngeneic mouse tumor model (LL/2). **(A)** A total of 5 x 10^4^ LL/2 cells were inoculated subcutaneously into the right flank of 14 week old female CB6F1 mice (10 mice group) on Day 0. Mice were dosed IP with 3 mkd anti-TeTx, anti-PD-L1 or anti-PD-L1-IFN-α on days 7, 11 and 15 post inoculation. Tumor length (L) and width (W) were measured via electronic caliper and the volume was calculated according to the following equation: V = L x W2/2 using Study Director version 3.1 (Studylog Systems, Inc., South San Francisco). **(B)** A total of 5 x 10^4^ LL/2 cells were inoculated subcutaneously into the right flank of 14 week old female CB6F1 on Day 0. Mice were dosed IP with 3 mkd anti-TeTx, anti-PD-L1 or anti-PD-L1-IFN-α on days 12. Two days after, tumors from each group were harvested and disaggregated. RNA was purified from the tumor samples analyzed for IP-10 qPCR. **(C)** CB6F1 mice were treated with the indicated doses of the fusion proteins and the greatest percentage of weight loss, relative to the initial weight of the cage of mice, is recorded. Animals were monitored once daily for adverse event. (**D)** CB6F1 mice were dosed IP with the antibody or fusion proteins and the blood samples were collected at the indicated time points for IP-10 ELISA.

IFN-α can increase the immunogenicity of tumor cells by up-regulation of MHC class I/tumor antigen complex and recruiting effector T cells via IP-10 induction. These mechanisms could potentially enhance the efficacy of anti-PD1/PD-L1 blockade and we chose to evaluate the efficacy of our anti–PD-L1-mIFN-α in a syngeneic mouse tumor model LL/2 that is nonresponsive to anti-PD-L1 treatment. Mice were dosed IP with anti-TeTx, anti-PD-L1 or anti-PD-L1-IFN-α on days 7, 11 and 15 post inoculation and anti–PD-L1-IFNα exhibited a very potent efficacy against LL/2 mouse tumors which was nonresponsive to anti-PD-L1 treatment (Fig 4A). Consistently, anti-PD-L1-IFN-α induced more IP-10 production in the LL/2 mouse tumors than the anti-PD-L1 or control anti-TeTx antibody (Fig 4B). Mice treated with the IFN-α fusion proteins had no significant weight loss or adverse event (Fig 4C). IFN-α fusion protein treatment induced a systemic IP-10 release at 6 hr after dosing and the IP-10 level declined at the 24 hr and the later time point (Fig 4D). These observations suggested that the IFN-α fusion antibodies could be well tolerated *in vivo*. Our data suggested that treatment of anti-PD-L1-IFN-α fusion protein is effective in an anti-PD-L1 resistant syngeneic mouse tumor model and the upregulation of IP-10 in the tumor microenvironment may contribute to the anti-tumor efficacy.

## Discussion

Treatments of IFNs were reported to correlate with clinical immune responses against cancer [17]. However, systemic delivery of type 1 IFNs is associated with toxicity thereby limiting clinical application [18]. To overcome this limitation, attempts have been made to generate IFN fusions proteins, including anti-HER2-IFN-α and anti-CD20-IFN-α fusion which demonstrated an improved therapeutic index and preclinical efficacy in murine and human lymphoma models [7, 19]. The use of IFN-β fusions to overcome resistance of anti-EGFR antibody by revitalizing innate and adaptive immune cells inside the tumor has also been proposed [5]. In the current study, we showed that targeting IFN-α to the cancer cells using an anti-PD-L1 antibody can be a novel strategy to overcome the resistance to PD-L1 blockade. Unlike lymphoma models where IFN-α can exhibit a direct tumor killing effect [20, 21], the anti-tumor activity of anti-PD-L1-IFN-α observed in this study were mostly immune related since it did not directly inhibit the growth of the mouse cancer cell line *in vitro* (S4 Fig). Here we observed that tumor targeted IFN-α induced selective IP-10 production which was critical for recruitment of effector T cells. Additionally, IFN-α upregulated MHC class I in tumor cells, which can enhance sensitivity to antigen specific T cell killing. IFN-α potentiates a distinct anti-tumor immune mechanism from anti-PD-L1 blockade and may explain why anti-PD-L1-IFN-α fusion can overcome the anti-PD-L1 resistance in the mouse model.

Liang Y et al recently reported an anti-PD-L1-mIFN-α fusion with mIFN-α fused to the N terminal of a human IgG Fc [21]. The fusion protein demonstrated efficacy to overcome PD-L1 blockade resistance in advanced mouse tumors, which was consistent with our observations in the current study. Instead of fusing mIFN-α to the N terminal of a human IgG Fc, we fused mIFN-α to the C terminal of a mouse IgG heavy chain (Fig 1A and 1B) and this approach has been shown to significantly decrease the IFN-α activity in a control fusion that does not engage tumor antigen [4], thereby limiting the potential off target toxicity of the fusion protein. Although only modest side effects were observed in mice treated with the fusion protein with mIFN-α fused at N terminal [21], the IFN-α receptor downstream signaling and its activity on Th1 T cell development differs between mice and humans [22], therefore more detailed analyses are required to understand the translational significance of these results.

Although Liang Y et al reported potent *in vivo* anti-tumor efficacy of the anti-PD-L1-mIFN-α fusion, the IFNR downstream pathways in the tumor cells that mediated the anti-tumor efficacy were not fully investigated. In the current study, we developed several *in vitro* assays to address the mechanisms of action of the anti-PD-L1-mIFN-α in the IFNR pathway of the tumor cells. First, we demonstrated that targeted IFN fusions (PD-L1 and EGFR) selectively induced IP-10 secretion from antigen positive tumor cells, which was critical in recruiting the effector T cells to the tumor microenvironment. Second, we developed an antigen-specific T cell killing assay to evaluate IFN-α fusion activity in enhancing CTL response. With this assay we demonstrated that anti-PD-L1-IFN-α at low concentration (1 pM) potentiated OT-I T cells killing against OVA expressing tumor cell lines while control IFN-α fusion did not show a similar effect. Anti-PD-L1-IFN-α increased MHC class I and tumor antigen complex expression and therefore could potentially sensitize the tumor cells for CD8^+^ T cell killing. Taken together, targeted IFN-α enhanced the immunogenicity of PD-L1 positive cancer cells and therefore may overcome the resistance to PD-L1 blockade in the mouse tumor models.

In summary, our current study demonstrated that targeting of IFN-α through anti-PD-L1 moiety can be an effective strategy in overcoming tumor resistance to PD-L1 blockade. Anti-PD-L1-mIFN-α induced MHC class I up-regulation on tumor cells which can enhance anti-tumor CD8^+^ T cell effector response. Targeting IFN-α to the tumor microenvironment may selectively induce IP-10 secretion from antigen positive tumor cells, enhance T cell infiltration and the effector T cell function for anti-tumor immune response. Therefore, our IFN-α fusion approach combined with other published data [12, 21, 23, 24] suggests that further evaluation of this approach for overcoming immunosuppression and increasing patient responsiveness to PD-L1 blockade is warranted.

## Supporting information

S1 Fig IFN fusions and recombinant mIFN-α induced IP-10 secretion from LL/2 cells. LL/2 cells were treated with recombinant mIFN-α or different mIFN fusions at the indicated concentrations for 72 hours and the IP-10 concentration in the supernatant was measured using IP-10 ELISA assay.
(TIF)

S2 Fig IFN-α fusions showed no activity in OT-1 T cell-mediated killing of OVA negative parental MC38 cell line. Two thousand MC38 cells were seeded in 96 well plates overnight. The next day, six thousand murine OT1 CD8^+^ T cells or naïve C57B CD8^+^ T cells were added. Co-cultured cells were treated with the indicated fusion proteins or antibodies for 5 days followed by a Cell Titer Glo assay.
(TIF)

S3 Fig IFN-α fusions and recombinant mIFN-α enhance antigen-specific T cell-mediated killing of tumor cells at different concentrations. Two thousand MC38-OVA or LL/2-OVA cells were seeded in 96 well plates overnight. The next day, six thousand murine OT1 CD8^+^ T cells were added. Co-cultured cells were treated with different concentrations of anti-PDL1-IFN-α, anti-TeTx-IFN-α, anti-PDL1, recombinant mIFN-α or media only for 5 days followed by a Cell Titer Glo assay.
(TIF)

S4 Fig IFN-α fusions showed no activity in inhibiting the proliferation of LL/2 cells *in vitro*. LL/2 cells were treated with different mIFN fusions at the indicated concentrations for 72 hours and the cell viability was measured using CellTiter-Glo assay.
(TIF)
