# Supplementary figures and images for: Empowering therapeutic antibodies with IFN-α for cancer immunotherapy
Source: PLoS One. 2019 Aug 8;14(8):e0219829. doi: 10.1371/journal.pone.0219829 (PMC6687177; doi:10.1371/journal.pone.0219829)

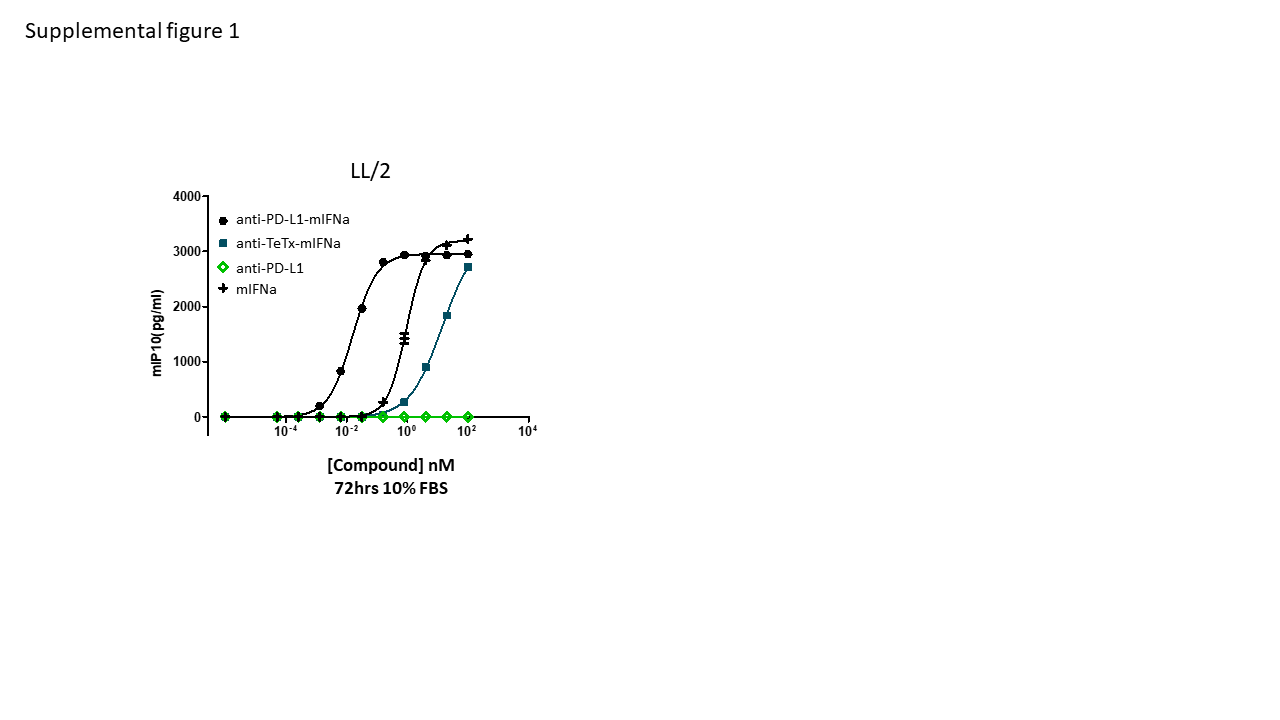

Supplement: S1 Fig — LL/2 cells were treated with recombinant mIFN-α or different mIFN fusions at the indicated concentrations for 72 hours and the IP-10 concentration in the supernatant was measured using IP-10 ELISA assay. (TIF) [file pone.0219829.s001.tif]

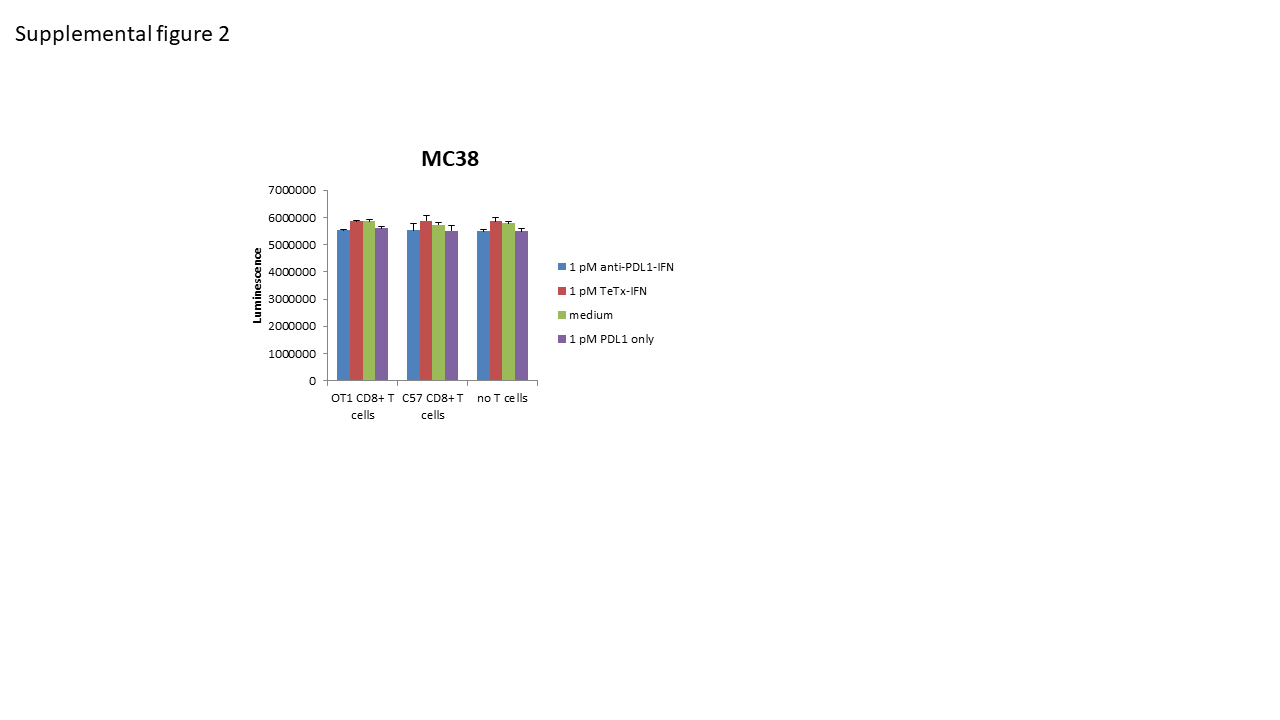

Supplement: S2 Fig — Two thousand MC38 cells were seeded in 96 well plates overnight. The next day, six thousand murine OT1 CD8+ T cells or naïve C57B CD8+ T cells were added. Co-cultured cells were treated with the indicated fusion proteins or antibodies for 5 days followed by a Cell Titer Glo assay. (TIF) [file pone.0219829.s002.tif]

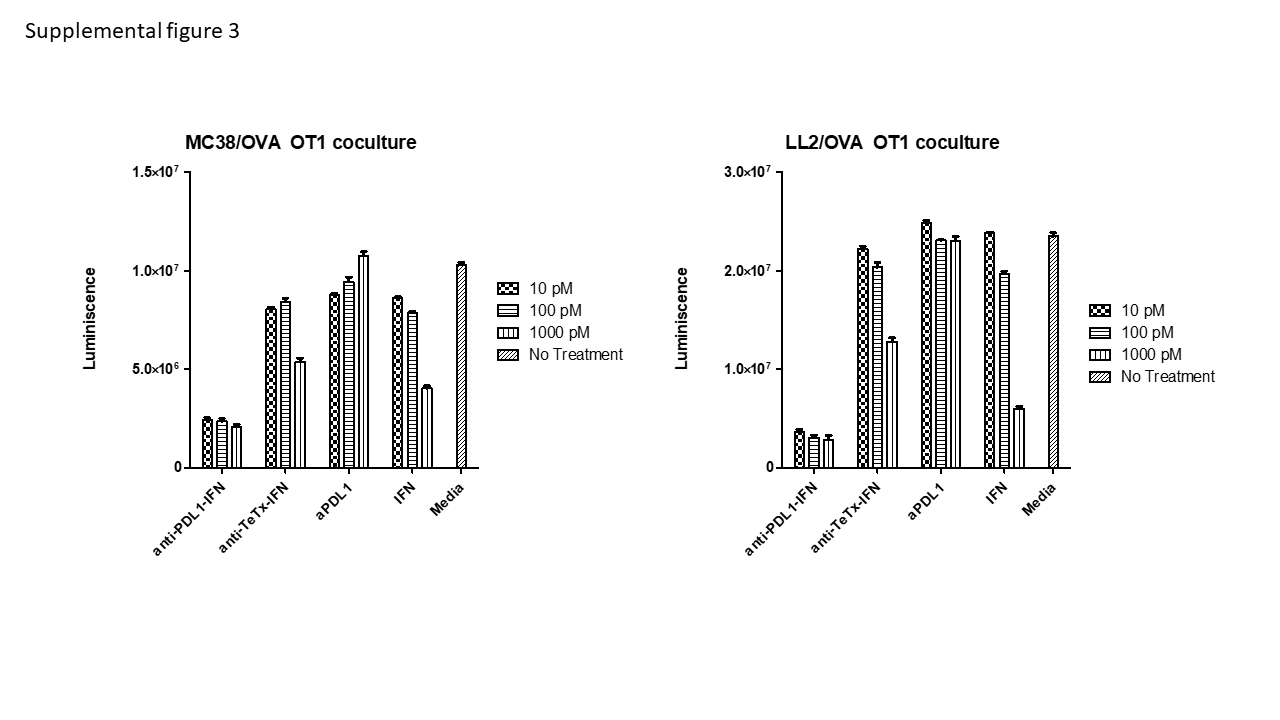

Supplement: S3 Fig — Two thousand MC38-OVA or LL/2-OVA cells were seeded in 96 well plates overnight. The next day, six thousand murine OT1 CD8+ T cells were added. Co-cultured cells were treated with different concentrations of anti-PDL1-IFN-α, anti-TeTx-IFN-α, anti-PDL1, recombinant mIFN-α or media only for 5 days followed by a Cell Titer Glo assay. (TIF) [file pone.0219829.s003.tif]

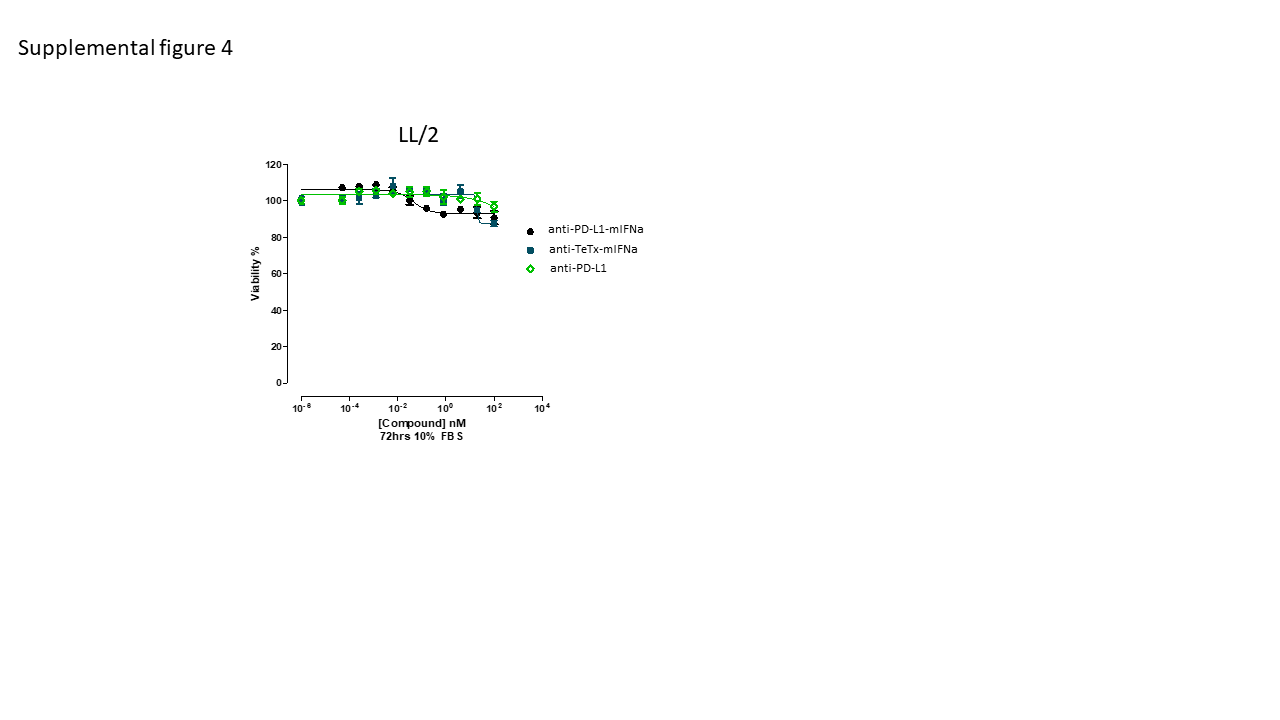

Supplement: S4 Fig — LL/2 cells were treated with different mIFN fusions at the indicated concentrations for 72 hours and the cell viability was measured using CellTiter-Glo assay. (TIF) [file pone.0219829.s004.tif]
